# Clinical practice guidelines for the management of chronic musculoskeletal pain in primary healthcare: a systematic review

**DOI:** 10.1186/s13012-016-0533-0

**Published:** 2017-01-05

**Authors:** Dawn V. Ernstzen, Quinette A. Louw, Susan L. Hillier

**Affiliations:** 1 Division of Physiotherapy, Faculty of Medicine and Health Sciences, Stellenbosch University, PO Box 241, Cape Town, 8000 South Africa; 2 Sansom Institute for Health Research, University of South Australia, Adelaide, 5000 Australia

**Keywords:** Clinical practice guidelines, Chronic musculoskeletal pain, Primary health, Systematic review

## Abstract

**Background:**

Up-to-date, high quality, evidence-based clinical practice guidelines (CPGs) that are applicable for primary healthcare are vital to optimize services for the population with chronic musculoskeletal pain (CMSP). The study aimed to systematically identify and appraise the available evidence-based CPGs for the management of CMSP in adults presenting in primary healthcare settings.

**Methods:**

A systematic review was conducted. Twelve guideline clearinghouses and six electronic databases were searched for eligible CPGs published between the years 2000 and May 2015. CPGs meeting the inclusion criteria were appraised by three reviewers using the Appraisal of Guidelines Research and Evaluation (AGREE) II.

**Results:**

Of the 1082 records identified, 34 were eligible, and 12 CPGs were included based on the inclusion and exclusion criteria. The methodological rigor of CPG development was highly variable, and the median domain score was 66%. The median score for stakeholder involvement was 64%. The lowest median score was obtained for the domain applicability (48%). There was inconsistent use of frameworks to aggregate the level of evidence and the strength of the recommendation in the included CPGs. The scope and content of the included CPGs focussed on opioid prescription.

**Conclusion:**

Numerous CPGs that are applicable for the primary healthcare of CMSP exists, varying in their scope and methodological quality. This study highlights specific elements to enhance the development and reporting of CPGs, which may play a role in the uptake of guidelines into clinical practice. These elements include enhanced reporting of methodological aspects, the use of frameworks to enhance decision making processes, the inclusion of patient preferences and values, and the consideration of factors influencing applicability of recommendations.

**Trial registration:**

PROSPERO CRD42015022098.

**Electronic supplementary material:**

The online version of this article (doi:10.1186/s13012-016-0533-0) contains supplementary material, which is available to authorized users.

## Background

Chronic musculoskeletal pain (CMSP) is a global healthcare concern. The condition is classified as a part of chronic non-malignant pain, which encompasses musculoskeletal, neuropathic and visceral pain, and pain from sickle cell disease [1]. CMSP negatively impacts physical and psychosocial health, daily function, participation in life roles, healthcare utilization, health-related quality of life [2–4]; and its management is associated with high financial, personal and resource costs. This complex condition involves biological, psychological, social and environmental factors. Therefore, a multidisciplinary and holistic approach to the management of CMSP is appropriate [5].

The prevalence of chronic pain is high and increasing [2, 6, 7]. A large proportion of individuals with CMSP present in primary healthcare settings for management [3, 8]. Primary healthcare settings appears to be ideally situated to deliver holistic care for the patient with chronic pain, addressing the health needs within the community [9], while integrating preventative, promotive, curative, and rehabilitation services. The components of primary healthcare are congruent with the proposition that chronic pain management should be multimodal and include rehabilitative options [1, 3, 10]. It is therefore vital that primary healthcare settings are adequately resourced to deal with the service-provision load associated with the holistic management of CMSP [3, 11]. These resources may include human resources, healthcare system, and policy and information resources.

Access to high quality, evidence-based, and up-to-date information resources can assist clinicians in making decisions about the care of patients with CMSP. Clinical practice guidelines (CPG) are one way of providing information on evidence-based and person-centred options for CMSP care. CPGs provide systematically developed clinical recommendations derived from best available evidence to guide clinicians and patients in making decisions about healthcare for specific clinical circumstances [12, 13]. CPGs may support decision-making by managers and policy-makers about the organization and delivery of healthcare [12, 14]. The implementation of CPGs can optimize healthcare by improving the quality, consistency, appropriateness, and cost-effectiveness of care [15–18]. Furthermore, CPGs play a role in enhancing provider and patient satisfaction with care [18]. CPGs are therefore useful means to enhance quality healthcare for the patient with CMSP by implementing evidence-informed management. Ideally, CPGs about CMSP should include a multidisciplinary perspective to facilitate the holistic management of CMSP.

The validity of CPGs depends on how well they were designed and conducted [14]. Clinicians often find it difficult to judge a CPG as trustworthy or of good quality due to the large number of CPGs available and the often contradictory information in them [13, 17]. In a good quality CPG, the clinical recommendations are rigorously developed, based on current, relevant scientific evidence, and it considers the benefits and harms of different healthcare options [13, 17, 19, 20]. Good quality CPGs encourage the use of interventions with proven benefits and discourages the use of ineffective or harmful interventions [21]. The cost-effectiveness of implementation, context-specific factors, and stakeholder involvement are important elements to be considered by a good CPG [20]. Despite the above quality indicators, several authors found the methodological quality of CPGs to be highly variable [16, 18, 20]. Consequently and several criteria have been developed to evaluate the methodological quality of CPGs, for example, the AGREE (Appraisal of Guidelines Research and Evaluation) instrument [13, 20, 22].

High quality, evidence-based, and up-to-date CPGs that are applicable for primary healthcare are crucial to optimize care for CMSP [7]. The multidimensional nature of CMSP, together with its associated burden, heightens the need for multimodal management strategies to prevent and manage the condition. A CPG is an important information resource to facilitate decision-making about care and to translate research findings into clinical practice. When considering the development of a CPG for the management of CMSP in primary healthcare, one needs to consider if such CPGs already exist, if existing CPGs are of high quality and if it represents the holistic and multimodal management for CMSP. The de novo development of CPGs can be expensive and time-consuming. Consequently, it has become a viable option to adopt, adapt, or contextualize existing CPGs in resource-constrained environments [23]. CPG contextualization refers to the consideration of possible differences in contextual factors such as personal and environmental features that may differ from one setting to the other. Knowledge about the existence and quality of CPGs for CMSP in primary healthcare, can inform the choice between adoption, adaptation, contextualization and de novo development of CPGs. The aim of this systematic review was therefore to methodically identify and appraise the available evidence-based CPGs for the management of adults with CMSP in primary healthcare settings.

The primary objectives were to:

- identify profession-specific or inter-professional CPGs on the management of CMSP in primary healthcare settings;
- critically appraise the quality of the included CPGs using the AGREE II instrument;
- determine the currency of the CPGs; and to
- determine the grading systems used to demarcate the level of evidence and the strength of the recommendation in the CPGs.

## Methods

### Study criteria

The study criteria were formulated using the PIPOH format (population, intervention, professions, health outcomes and health setting) for guideline reviews [24]. The types of studies eligible were CPGs available in full text and published between the years 2000 and May 2015. The date cut-off was set to ensure up-to-date CPGs. The target population and disease characteristic of the CPGs included adults with CMSP. It was anticipated that recommendations for CMSP would be included in CPGs for chronic non-malignant pain. CPGs that focused exclusively on chronic pain from non-musculoskeletal origin such as sickle cell disease, neuropathic pain, and malignancy was excluded due to the differences in pathology, pain mechanisms, and possible management strategies.

The types of interventions could include evaluation, diagnosis, and management of CMSP. Examples of such interventions include inter-professional interventions, pharmacological, and non-pharmacological management, rehabilitative options, and self-management. CPGs that targeted any healthcare professionals involved in the management of CMSP were eligible for inclusion. The expected outcomes of CPGs could include patient outcomes, system outcomes, or public health outcomes. For the purpose of this review, only CPGs focused on primary healthcare settings were included. CPGs that focussed solely on secondary, tertiary, or specialist healthcare settings were excluded due to different management options offered.

### Search strategy

The primary investigator searched the electronic databases of the following guideline clearinghouses: the US National Guideline Clearinghouse (US NGC); Agency for Healthcare Research and Quality (AHRQ); Guidelines International Network (G-I-N); Scottish Intercollegiate Guidelines (SIGN); United Kingdom’s National Institute for Health and Clinical Excellence (NICE); New Zealand Guidelines Group (NZGG); WHO guidelines; TRIP database; National Institutes of Health (NIH); Monash University Centre for Clinical Effectiveness; Australia’s National Health and Medical Research Council (NHMRC); Canadian Medical Association Clinical Practice Guidelines Infobase, and the Institute for Clinical Systems Improvement (ICSI). In addition, the following online databases were searched to include those guidelines that were peer reviewed and published in journals: CINAHL, PEDro; PubMed, EBSCO host, and Medline. The broad search terms included: clinical practice guidelines; OR clinical guidelines; OR care pathway; OR clinical pathway; OR care protocol; AND chronic pain; OR chronic musculoskeletal pain; OR chronic non-malignant pain; OR chronic non-cancer pain; AND adults; AND primary care; OR primary healthcare.

The initial search was conducted from July to October 2014, and the search was updated during May 2015. The inclusion and exclusion criteria were applied by screening the identified CPG titles and objectives to select the eligible CPGs. The data was extracted in the PIPOH format to enable this analysis. The process was verified by the co-authors on a random audit basis. A record of search yields and decision-making was kept.

### Data extraction

The primary investigator extracted the following data into custom built data extraction sheets:

- The clinical question formulation using the PIPOH format.
- Guideline currency: the publication date of the CPG and periods covered by the literature search in the guideline, date of revision.
- General information: the developing organization/authors; country of publication; language of publication.

### Critical appraisal

The methodological quality of eligible CPGs was independently assessed by three reviewers using the AGREE II instrument [25]. The AGREE II is an internationally developed, widely accepted, valid, reliable, easy to use, and transparent instrument to assess the methodological rigor of the reported guideline [18, 22]. It contains 23 key quality items categorized in six domains, scored on a 7-point Likert scale. The AGREE II evaluates the process of CPG development and the quality of reporting. However, it does not evaluate the content of the CPG, nor the quality of evidence supporting the recommendations. Each AGREE II domain focuses on a separate aspect of guideline quality, namely, scope and purpose, stakeholder involvement, rigor of development, clarity of presentation, applicability, and editorial independence.

The reviewers’ AGREE scores were entered into a custom built Excel spreadsheet by the primary investigator. Any difference in score higher than two points was discussed amongst the project team to reach consensus. A quality score was calculated for each of the six AGREE II domains using the guiding principles and the following formula provided in the user manual:

Domain score = (obtained score–minimum possible score)/(maximum possible score–minimum possible score) × 100 = %. All item scores in a domain were summed, and the total was standardized as a percentage of the maximum possible score for that domain. The median domain score and interquartile range (IQR) for each domain was then calculated [26].

## Results

### Search results

**Fig. 1:**
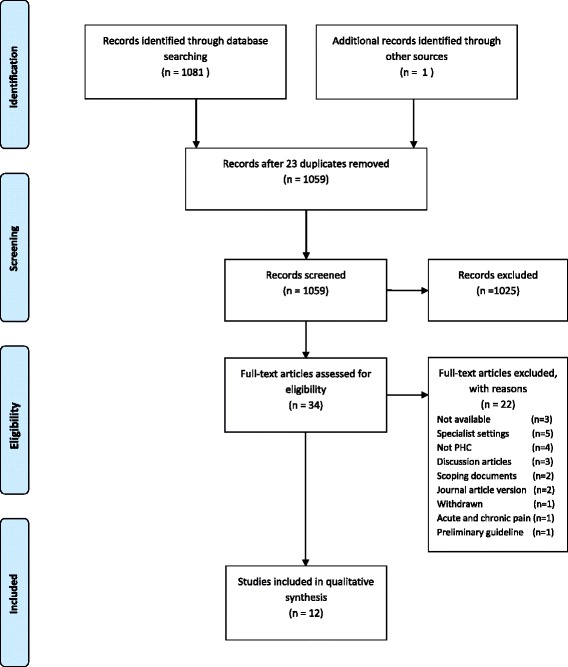
Diagram of search results (PRISMA format)

The results of the systematic search are summarized in Fig. 1. The search yielded two categories of CPGs, namely, comprehensive CPGs and CPGs that were published in journal article format. Where indicated, contact was made with the authors to obtain full text CPGs. Thirty-four eligible CPG were considered for inclusion. After applying the inclusion/exclusion criteria, 12 CPGs were included. The main reasons for exclusions are summarized in Fig. 1.

### Included clinical practice guidelines

Table 1 summarizes information about the included CPG and their currency. One of the included CPGs originated from a middle-income country (CPG 10). Six out of the 12 included CPGs focused on the prescription of opioids (CPGs 1, 3, 6, 7, 8, and 10), and two focused specifically on the management of musculoskeletal pain (CPG 9 and 12).

**Table 1:** Included CPGs (*n* = 12)

CPG number	Title of guideline	Organization/authors	Country of origin	Guideline currency		
				Date for revision	Publication date	Search dates covered
CPG 1	Guidelines for responsible opioid prescribing in chronic non-cancer pain: Part 1—evidence; Part 2—guidance	American Society of Interventional Pain Physicians (ASIPP) [41]	USA	June 2015	2012 (update on 2008 version)	Not stated
CPG 2	Assessment and management of chronic pain	Institute for Clinical Systems Improvement (ICSI) [31]	USA	Every 24 months, i.e., December 2015	2013 (update on 2011 version)	August 2011–August 2013
CPG 3	Canadian guideline for safe and effective use of opioids for chronic non-cancer pain	National Opioid Use Guideline Group (NOUGG) [42]	Canada	Not stated	2010	2005–July 2009
CPG 4	Assessment and management of pain	Registered Nurses’ Association of Ontario (RNAO) [32]	Canada	Every 5 years, i.e., 2018	2013 (update on 2002, 2007 versions)	2007–2012
CPG 5	Management of chronic pain: a national clinical guideline.	Scottish Intercollegiate Guidelines Network (SIGN) [30]	UK	In 3 years, i.e., 2016	2013	2007–2012
CPG 6	Managing chronic non-terminal pain in adults including prescribing controlled substances	University of Michigan Health System (UMHS) [43]	USA	Not stated (previous was 2009, 2011)	2011	1995–2010; (search dates 1998 –2002)
CPG 7	Interagency guideline on opioid dosing for chronic non-cancer pain: an educational aid to improve care and safety with opioid treatment	Washington State Agency Medical Directors’ Group (WSAMDG) [44]	USA	Not stated	2010 (update on 2007 version)	Not stated
CGA 8	Clinical guidelines for the use of chronic opioid therapy in chronic non-cancer pain	The American pain society (APS)–American academy of pain medicine (AAP) [45]	USA	2012	2009	Through to July 2008 (not stated from when)
CGA 9	Managing musculoskeletal complaints with rehabilitation therapy: summary of the Philadelphia panel evidence-based clinical practice guidelines on musculoskeletal	The Philadelphia Panel Members and Ottawa Methods Group [46, 47]	Canada and USA	Not stated	2002	Through to July 2000
CGA 10	South African guideline for the use of chronic opioid therapy for chronic non-cancer pain	Raff M, Eppel S, Meyer H, Sarembock B, Webb D [48]	SA	Not stated	2014	Not stated four existing CPGs published between 2009–2012 were chosen (CPG 1, 2, 8 in this Table)
CGA 11	Evidence-based clinical practice guidelines for interdisciplinary rehabilitation of chronic non-malignant pain syndrome patients	Sanders SH, Harden NR, Vicente PJ [33]	USA	Every 4 years	2005 (update on 1995, 1999 version)	September 1999 (end date not stated)
CGA 12	Update on guidelines for treatment of chronic musculoskeletal pain	Schnitzer TJ [7]	USA	Not stated	2006	Not stated

(*CPG* clinical practice guideline, *CGA* clinical guideline in article format, *USA* United States of America, *UK* United Kingdom, *SA* South Africa)

### Methodological quality

The AGREE II domain scores (median and range) are provided in Table 2. The domains with the lowest median score were domain 2 (stakeholder involvement), domain 3 (rigor of development), and domain 5 (applicability). Further analysis of each question within a domain is represented in Table 3. Additional file 1 provides more information on the guideline development group and the stated scope for each CPG.

**Table 2:** Combined scores of the three reviewers in %

	CPG 1	CPG 2	CPG 3	CPG 4	CPG 5	CPG 6	CPG 7	CPG 8	CPG 9	CPG 10	CPG 11	CPG 12	Median	IQR
Domain 1: scope and purpose	89	96	98	80	93	48	89	76	67	78	63	48	79	24
Domain 2: stakeholder involvement	65	85	91	70	100	52	63	80	57	35	22	15	64	34
Domain 3: rigor of development	72	85	94	88	94	47	40	84	60	30	51	30	66	41
Domain 4: clarity of presentation	91	89	87	96	98	87	69	90	83	69	57	72	87	19
Domain 5: applicability	60	79	75	92	79	53	32	43	21	14	15	7	48	57
Domain 6: editorial independence	97	100	100	67	67	97	44	100	25	100	0	86	91.5	39
TOTAL	474	534	545	493	531	384	337	473	313	326	208	258		
Overall score out of 7	5	5.6	6	5.3	6.5	4	3.6	5	3.6	3	3	3		

*CPG* clinical practice guideline

**Table 3:** All domain scores

	CPG 1	CPG 2	CPG 3	CPG 4	CPG 5	CPG 6	CPG 7	CPG 8	CPG 9	CPG 10	CPG 11	CPG 12	Total	Percentage (%)
Domain 1: scope and purpose														
1. Overall objectives specific	21	20	21	17	18	18	19	17	15	16	13	14	209	83
2. Health questions specific	18	20	21	19	21	9	18	16	14	15	13	9	193	77
3. Population is specific	18	21	20	16	20	8	20	17	16	20	17	12	205	81
Domain 2: stakeholder involvement														
4. Guideline development group	19	18	20	15	21	15	18	19	20	7	6	10	188	75
5. Views and preferences of target population	5	21	18	11	21	6	7	14	2	4	3	3	115	46
6. Target users clearly defined	20	16	20	21	21	16	18	19	18	17	12	4	202	80
Domain 3: rigor of development														
7. Systematic methods	15	17	21	21	21	19	10	13	12	6	16	4	175	69
8. Criteria for evidence selection	14	19	21	21	21	17	5	15	19	7	16	4	179	71
9. Strengths and limitations of evidence	13	19	20	18	18	6	5	20	19	10	9	4	161	64
10. Methods for formulating recommendations	19	17	21	18	19	8	10	21	17	5	8	11	174	69
11. Health benefits, side-effects, risks were considered in formulating recommendations	20	19	19	17	19	19	21	21	9	18	12	20	214	85
12. Explicit link between recommendations and evidence	19	17	21	20	21	12	10	19	17	11	12	12	191	76
13. Externally reviewed prior to publication	15	18	21	16	21	6	16	18	15	7	10	9	172	68
14. A procedure for updating guideline	13	20	15	20	19	5	4	18	3	3	14	3	137	54
Domain 4: clarity of presentation														
15. Recommendations specific; unambiguous	20	19	21	20	20	18	17	20	17	15	13	18	218	87
16. Different options for management	18	19	14	21	21	20	13	18	18	15	15	16	208	83
17. Key recommendations easily identifiable	20	19	21	20	21	18	16	20	19	14	12	14	214	85
Domain 5: applicability														
18. Barriers and facilitators to application	15	16	19	20	17	11	6	8	11	4	4	5	136	54
19. Advice/Tools to put recommendations to practice	14	20	21	20	18	8	13	10	9	7	8	6	154	61
20. Potential resource limitations have been considered	9	13	12	18	15	11	7	12	4	3	3	3	110	44
21. Monitoring and audit criteria	15	20	14	20	19	20	9	13	3	8	8	3	152	60
Domain 6: editorial independence														
22. Views of funding body vs guideline content	20	21	21	15	10	20	14	21	12	21	3	17	195	77
23. Competing interests of guideline development group members have been recorded and addressed	21	21	21	15	20	21	8	21	3	21	3	20	195	77

*CPG* clinical practice guideline

Domain score = (obtained score–minimum possible score)/(maximum possible score–minimum possible score) × 100 = % [25]

CPGs consistently inadequately adhered to topic 5 in domain 2 (views and preferences of target population). Only 3 of the 12 included CPGs reported that they sought patient perspectives as part the CPG development. The methods used in these CPGs included focus groups with patients (CPGs 3 and 2); patients as part of the guideline development group (CPG 5); a literature search of patients’ preferences (CPG 5); an environmental scan through surveys, key informant interviews and focus groups (CPG 2). The strengths and limitations of evidence (topic 9), was also inconsistently adhered to. Within domain 3 (rigor of development), topic 14 had the lowest scores, indicating that few CPGs included a procedure for updating the CPG. All four topics in domain 5 (applicability) were challenging for the CPG developers to report on, as indicated by the low scores.

The analysis of domain 5 (applicability) indicated that algorithms and outcome measure tools for putting recommendations into practice was the most common strategies provided to facilitate implementation of CPG recommendations (see Additional file 2). Although guideline developers mentioned barriers and facilitators for the use of the CPG (CPG 1, 3, 4, 5); this aspect was not covered in-depth. The consideration of resource implications was partially covered. Policies, as well as monitoring and evaluation criteria for opioid therapy was a consideration that was well attended to by the CPGs focusing on opioid prescription (CPG 1, 2, 6, 7, and 8).

### Evidence grading systems used by the clinical practice guidelines

The CPGs used a variety of grading systems to categorize the levels of evidence and the strength of the recommendation. These grading systems are summarised in Table 4. Three CPGs did not grade the level of evidence or the strength of the recommendation. Four CPGs graded the level of evidence, but not the strength of the recommendation.

**Table 4:** Grading systems used to determine the level of evidence and the strength of the recommendation

CPG		Name of grading system	Level of evidence grading	Strength of recommendation
1	ASIPP [41]	United States Preventive Services Task Force (USPSTF) criteria	Good Fair Poor	None
2	ICSI [31]	In transition between ISCI system to GRADE. Thus, using an hybrid system	High quality evidence Low quality evidence	None
3	NOUGG [42]	–	I, II-1 II-2 II-3 OR III	A, B, C
4	RNAO [32]	Adapted SIGN	Ia Ib IIa IIb III IV	None
5	SIGN [30]	SIGN	1++, 1+ 2++; 2+; 2- 3 4	A, B, C, D, Good practice point
6	UMHS [43]	–	A B C D	I II III
7	WSAMDG [44]	Rating scheme (not provided)	Not provided	None
8	APS AAP [45]	Adapted GRADE methodology.	High quality evidence Medium quality evidence Low quality evidence	Strong recommendation Weak recommendation
9	Harris [46, 47]	Modified Canadian Task Force Grading System	I, II-1 II-2 II-3 OR III	A, B, C
10	Raff [48]	–	None	None
11	Sanders [33]	–	None	None
12	Schnitzer [7]	–	None	None

*CPG* clinical practice guideline

### Guideline content overview

Table 5 provides a content overview of the included CPGs. In CPG 7, 9, 10, 11, and 12, no clear recommendation statements were identifiable, and therefore an overview is presented. The writing style of these CPGs focused on a discussion of relevant information, and was less focused on making clear recommendations. The content of the CPGs focused on assessment of CMSP and on the prescription of opioids. Eight out of the 12 CPGs included at least one position statement about rehabilitation options for CMSP.

**Table 5:** Content section areas of the included CPG

Content section	CPG 1	CPG 2	CPG 3	CPG 4	CPG 5	CPG 6	CPG 7	CPG 8	CPG 9	CPG 10	CPG 11	CPG 12	Total	Percentage (%)
	ASIPP	ICSI	NOUGG	RNAO	SIGN	UMHS	WSA MDG^a^	APS-AAP	Harris^a^	Raff^a^	Sanders^a^	Schnitzer^a^		
Evaluation/assessment	√	√	√	√	√	√	√	√		√	√		10	83
Diagnosis	√	√				√		√		√	√		6	50
Planning of care	√	√		√	√	√		√		√	√		8	67
Complementary therapy		√			√								2	17
Diet therapy					√							√	2	17
Practitioner education				√									1	8
Occupational therapy					√						√		2	17
Organization and Policy				√									1	8
Pharmacological therapy (non-opioids)		√		√	√	√				√	√	√	7	58
Pharmacological therapy (opioids)	√	√	√	√	√	√	√	√		√	√	√	11	92
Physical therapy		√		√	√			√	√	√	√	√	8	67
Psychologically based therapy		√		√	√			√		√	√		6	50

*CPG* clinical practice guideline

^a^No clear recommendation statements

## Discussion

This systematic review, to the knowledge of the authors, is the first to focus on identifying and appraising profession-specific and inter-professional CPGs for the management of CMSP in the primary healthcare settings. One of the main findings was that there are multiple, current CPGs on the topic that had been developed by different organizations and authors. Considerable time, effort, and resources were therefore invested in the development of these CPGs. Two guidelines focus specifically on CMSP management in primary healthcare, and recommendations for the management of CMSP were imbedded in the CPGs for chronic non-malignant pain. CPGs were mainly profession-specific, few took a multidisciplinary perspective, and CPGs varied in their scope, coverage, format, and quality.

The scope of the majority of included CPGs focused on opioid prescription and congruently, the content of the CPGs is also focused on opioid prescription (Tables 1 and 5, Additional file 1). The focus on opioid prescription can be ascribed to the dramatic rise in the prescription of opioids, as a result of the increase in the prevalence of chronic pain and the increase in dosage and frequency of prescription [10, 27–29]. The risks associated with opioid use may have created a growing need for clinical guidance on decision-making for opioid prescription. It is thus likely that high quality, evidence-based CPGs became fundamental to provide guidance for the safe prescription of opioids for CMSP. In contrast, although included CPGs advocated non-pharmacological management options for the management of CMSP in primary healthcare, recommendations about these options were seldom included. SIGN [30], ICSI [31], and RNAO [32] provided multiple recommendations on various rehabilitation options, while Sanders et al. [33] and Schnitzer [7] mentioned multimodal options. The lack of focus of non-pharmacological management options was congruent with the inadequate representation of rehabilitation practitioners in the guideline development teams of included CPGs. Since several high quality CPGs are now available for the prescription of opioids, we recommend the future guidelines should include a holistic and multidisciplinary scope. The prevention and management of CMSP requires a holistic and inter-/multidisciplinary approach to address the complex interaction between biological, psychological, social and environmental factors [5]. We argue that a renewed scope on holistic management, with a congruently aligned multidisciplinary guideline development team, is needed for future CPGs on the primary healthcare of CMSP, to address the burden associated with the condition. 

The review revealed three AGREE II domains that require consideration to enhance the quality of future CPGs for CMSP in primary healthcare settings, namely, the domains of rigor of development, stakeholder involvement, and applicability. The findings are congruent with that of Shaneyfelt et al. [16] and Misso et al. [34]. Shaneyfelt et al. [16] reviewed 279 CPGs to determine adherence to quality standards. They identified aspects needing improvement in the reviewed CPG as: the identification of evidence, the formulation of recommendations, guideline expiry date, cost implications, and the role of values and preferences. Misso et al. [34] reviewed CPGs for osteoarthritis of the knee and hip and found stakeholder involvement, rigor of development, applicability. and editorial independence to be poorly addressed.

In our review, the median score for reporting of rigor of development (domain 3) was adequate; however, the range of these scores was wide. The criteria that presented challenges were the aggregation of the strengths and limitations of the body of evidence and updating the CPG. More than half of the included CPGs were current and were published 5 years prior to our systematic search. Guideline developers put little emphasis on providing procedures and dates for updating the CPGs, which was also a key deficiency found in Vernooij’s et al. [35] systematic review of guideline handbooks. Providing details about updating a CPG is important, because they may become outdated as new evidence for interventions become available, emphasizing the need for recommendations to be modified [13, 15]. We recommend that the domain of methodological rigor should receive detailed consideration during future CPG development to improve the credibility of the evidence base on which recommendations are built. Not adhering to the criteria of this domain may threaten the trustworthiness and consequently uptake of CPG recommendations in primary healthcare practice. A thorough, unbiased review and aggregation of the evidence may prevent incorrect or biased recommendations [14].

This systematic review highlights the inconsistent use of frameworks to aggregate the level or body of evidence and the strength of the recommendation by the included CPGs. The criterion of using a framework to rate the strengths and limitations of the body of evidence is evaluated in the AGREE II domain of methodological rigor (topic 9). The AGREE II considers formal or informal methods for this domain. A framework that guides the decision-making process for recommendations ensures transparency and objectivity [14] and provides logical methods for considering the entire body of evidence relevant to a particular clinical question. Determining the body of evidence that underpins a CPG recommendation is critical in the guideline development process. Without transparency of the framework used to aggregate the body of evidence, guideline users cannot determine whether recommendations are built on strong evidence or weak evidence [14]. Furthermore, few included CPGs used the body of evidence together with the strength of the recommendation to frame recommendations. Determining the strength of the recommendation guides the applicability and implementability of the recommendations. The strength of the recommendation is dependent on the quality of the evidence, the balance between desirable and undesirable effects, values and preferences of the stakeholders and cost of implementation [36]. During the development of FORM (Australian method for formulating and grading recommendations), several efficient frameworks for developing recommendations were identified [37]. These frameworks included GRADE (Grading of Recommendations Assessment, Development and Evaluation), SIGN (Scottish Intercollegiate Guidelines) and SORT (Strength-of-Recommendation Taxonomy) [37]. The frameworks consider the evidence base (study quality, size, precision of results, bias); the consistency of different study findings; the clinical impact based on the body of evidence; generalizability of the results; and the applicability to the context that it is intended for [37]. The process of guideline development is evolving, and recently, more emphasis has been placed on the decision-making process of moving from evidence to recommendations. The use of evidence to decision frameworks is advocated as a systematic and transparent process to formulate recommendations [38]. In the review, guideline developers reported to use formal or informal consensus processes to make decisions. However, the use of evidence to decision frameworks was not explicitly stated in the included CPGs (also not a criterion for AGREE II). CPGs using GRADE (see Table 4), may have used a framework to consider factors that influence the strength and direction of the recommendation. The use of frameworks or a writing guide to frame recommendations, to grade the body of evidence and to determine the strength of the recommendation is advocated as a way to ensure consistency and transparency when making decisions and writing recommendations. Guideline developers are encouraged to use frameworks such as FORM, GRADE, SIGN, and SORT to enhance systematic decision-making processes in CPG construction.

The inclusion of stakeholders in the development process of CPGs is assessed as part of AGREE II (domain 2), and adherence to this quality criterion is thought to enhance a sense of ownership, thereby facilitating the uptake of the guideline [19]. While most included CPGs representation from professional groups, guideline developers did not commonly include the views and preferences of the target populations (patients and public). Shaneyfelt et al. [16] and Misso et al. [34] likewise found that patient values and preferences were not effectively addressed during CPG development. Patient perspectives and preferences influences healthcare utilization, therefore, patient perspectives about CMSP and its treatment are important in CPG development [20]. Patient preferences and concerns are shaped by context factors, which in turn impact the formulation and applicability of guideline recommendations. Consequently, some recommendations in a CPG may be preference sensitive. The inclusion of patient perspectives in the guideline development process is an important step towards facilitating relevant, person-centred care, and autonomy [39].

The AGREE domain of applicability (relevance) considers context factors that may impact CPG implementation, namely, the facilitators, barriers, and resource implications for implementation; tools for putting recommendations into practice, as well as monitoring and auditing criteria that influence the uptake and implementation of a CPG. These criteria therefore represent the features of the healthcare system, resources, cultural, and organizational factors that should be considered in framing guideline recommendations [15, 17, 19, 36]. Gagliardi et al. [12] contends that information on implementability within a CPG may assist the end-users to adopt the recommendations. In this review, the domain of applicability received the lowest median score. It is plausible that context features were not included in the CPG document, but were a part of the implementation plan for the CPG in a particular setting. The RNAO [32] and SIGN [30] contend that each setting/institution may benefit from a tailor-made implementation plan. Participation of local stakeholders can ensure that the CPG and its recommendations are compatible with the local context. It is possible that explicit statements on implementation parameters were not included as they may differ from one setting to the other. Additionally, the use of evidence to decision frameworks considers population or patient-specific outcomes, including cost-effectiveness, applicability, feasibility, subgroups, and generalizability when framing recommendations [37, 38]. The use of these frameworks may provide useful information about the context and application of recommendations, which may enhance CPG quality and uptake, and inform processes such as CPG adoption, adaptation, and contextualization [23, 24]. Regardless of the use of frameworks, it is advisable that future CPG on CMSP include more information on potential resource implications and barriers and facilitators to implementation.

Transparency and specificity regarding context factors that may impact recommendations is becoming increasingly important due to the option of adopting, adapting, or contextualizing CPGs for different settings, when de novo development of CPGs is not a feasible option. Knowledge about the context sensitive recommendations, may inform the above processes of CPG development. It is therefore an option that future CPGs on CMSP highlights preference or context sensitive recommendations [39]; and link these recommendations to context applicable implementation strategies, since context factors may play a vital role in applicability. An example of signposting context sensitive recommendations, is illustrated in the novel concept of CPG contextualization, whereby context and practice points are produced and linked to relevant context sensitive recommendations [23, 40]. The inclusion of context and practice points is thought to enhance CPG the application of recommendations in practice.

CPGs can be an important information resource for the evidence-based and holistic management of CMSP and has the potential to influence policy and practice about CMSP care. Given the multidimensional nature of CMSP, it is important that the scope of future CPGs include a multidisciplinary approach, which implies that healthcare systems in which CMSP is managed should ideally allow for a multidisciplinary management approach. While some included CPGs elaborated on the cost and cost-effectiveness of interventions, the findings of this review indicate that there is great opportunity for CPGs to elaborate on cost and policy implications that guideline recommendations may have. The RNAO [32] included the potential impact of CPG recommendations on policy, legislation, healthcare systems, and health professions education for CMSP. One policy aspect that was well addressed in the CPGs was the need for policy on the prescription and monitoring of opioids for CMSP. The inclusion of information on policy and practice implications is useful and may play a role in the organizational acceptance and implementation of the CPG.

We identified several strengths in the CPGs, which included that guideline developers clearly communicated the overall objectives, scope, and focus of the CPG. The domain on clarity of presentation obtained a high score, indicating that CPGs presented their recommendations in a clear, easily identifiable, and specific format. Editorial independence had also been adhered to in most included CPGs.

### Implications for practice

This systematic review highlights the strengths and limitations of current CPGs on the management of CMSP in primary healthcare settings. Guideline developers who are considering adopting, adapting, contextualizing, updating existing CPGs, or developing new CPGs for CMSP should consider the findings of this systematic review to optimize the methodological quality and uptake of CPGs. Based on the findings of this review, the authors recommend that future CPGs should:

- Include decision-making frameworks for the level of evidence, strength of recommendation, and framing recommendations in the CPG development process.
- Include the relevant stakeholders and end-users in the process of CPG development.
- Consider and document barriers and facilitators that may influence the uptake of clinical guideline recommendations in the intended setting.
- Develop and document strategies to facilitate the successful uptake and implementation of CPGs into practice.
- Provide dates and procedures for updating the CPG.

We further recommend that, due to the many CPGs on the topic, existing high quality CPGs be contextualized to local circumstances, particularly in resource-constrained environments. In this way, resources may be used to further the uptake of CPG recommendations with a rigorous implementation and sustainability strategy, instead of resources being spent on de novo CPG development [23]. Where de novo development of CPGs are feasible and the focus should be on a multidisciplinary scope. We found that CPGs written in the article format had several limitations that can be attributed to the restrictions of the journal. We recommend that in the case of a journal format, supplementary material be made available about methodological development.

The similarities between the findings of this systematic review and that of Shaneyfelt et al. [16] and Misso et al. [34] indicate after more than a decade guideline developers are still finding it challenging to adhere to certain quality standards of developing CPGs. The reasons for this phenomenon warrant further investigation.

### Limitations

Our review had some limitations of its own. We applied the AGREE II instrument, which evaluates the reported rigor of development and not the content of the CPG. The next step in our process is the content analysis of high quality CPGs for CMSP in primary healthcare settings. Our focus was specific to primary healthcare settings, and this may have excluded some guidelines that are applicable in this setting, but not stated overtly to be so. The CPGs we identified were predominantly from developed countries, so the appropriateness of these guidelines in other countries is uncertain. Furthermore, we acknowledge that although we did an extensive literature search, we may have missed eligible CPGs.

## Conclusion

The systematic review found several CPGs that are applicable for the primary healthcare of CMSP. The included CPGs vary in their scope and methodological rigor. There was little emphasis on non-pharmacological strategies in the composition of the guideline development team, the stated scope, and the content of the CPGs.This study highlights specific elements for enhancement in the development and reporting of future CPGs, which may play a role in the uptake of CPGs into clinical practice. These elements include enhanced reporting of rigor of development, the inclusion of stakeholder preferences and values and the consideration of context specific factors (e.g., culture, healthcare setting). Further research on methods to include patient preferences and how this impacts CPG content and implementation is needed.

## Additional files

Additional file 1: Scope and development group members of CPGs. (DOCX 20 kb)
Additional file 2: More on DOMAIN 5: applicability. (DOCX 17 kb)

## Abbreviations

AAP: American academy of pain medicine
AGREE II: Appraisal of guidelines research and evaluation, version II
APS: The American pain society
ASIPP: American society of interventional pain physicians
CMSP: Chronic musculoskeletal pain
CGA: Clinical guideline in article format
CPG: Clinical practice guideline
FORM: An Australian method for formulating and grading recommendations in evidence-based clinical guidelines
GRADE: Grading of recommendations assessment, development and evaluation
ICSI: Institute for clinical systems improvement
IOM: Institute of medicine
IQR: Interquartile range
NICE: United Kingdom’s National Institute for Health and Clinical Excellence
NOUGG: National opioid use guideline group
PIPOH: Population, intervention, professions, health outcomes, and health setting
RNAO: Registered nurses’ association of Ontario
SIGN: Scottish intercollegiate guidelines
SORT: Strength-of-recommendation taxonomy
UK: United Kingdom
UMHS: University of Michigan health system
USA: United States of America
USPSTF: United States preventive services task force
WHO: World Health Organization
WSAMDG: Washington state agency medical directors' group
