# Supplementary material for: Clinical practice guidelines for the management of chronic musculoskeletal pain in primary healthcare: a systematic review
Source: Implement Sci. 2017 Jan 5;12:1. doi: 10.1186/s13012-016-0533-0 (PMC5217556; doi:10.1186/s13012-016-0533-0)
Supplement: Additional file 1: — Scope and development group members of CPGs. (DOCX 20 kb) [file 13012_2016_533_MOESM1_ESM.docx]

### Additional file 1: Scope and development group members of CPGs

|  | **Nr of development group members** | **Professions included** | **Stated objective/scope of CPG** |
| --- | --- | --- | --- |
| **CPG 1 (ASIPP)** | 55 | 2 pharmacists, 2 psychologists, 2 registered nurses, 1 statistician, 1 physical therapist, 2 research coordinators, 1 librarian, 1 academic radiologist, 3 residents or fellows, and the remaining (40) were practicing interventional pain physicians, either in an academic setting or in private practice | To provide guidance for the use of opioids for the treatment of chronic non-cancer pain, to produce consistency in the application of an opioid philosophy among the many diverse groups involved, to improve the treatment of chronic non-cancer pain, and to reduce the incidence of abuse and drug diversion. The focus of these guidelines is to curtail the abuse of opioids without jeopardizing non-cancer pain management with opioids. |
| **CPG 2 (ICSI)** | 12 working group members | 1 clinical psychologist; 3 pharmacists; 7 medical doctors or varying specialities; 2 unclear | The guideline will address the management of chronic pain for adults. It can be applied to paediatric populations where noted. It is not intended for the treatment of migraine headaches, cancer pain, advanced cancer pain, or in the context of palliative care or end-of-life management. Topics of addiction, withdrawal, tapering or methadone are not inclusively addressed within the context of this guideline. |
| **CPG 3 (NOUGG)** | Three groups were involved in developing the Canadian Guideline: National Opioid Use Guideline Group (NOUGG), Research Group, and National Advisory Panel (NAP). | NOUGG is a task-specific group (19 members) (1 pharmacist)  The Research group comprised six members: a physician/epidemiologist, four physician-researchers, and a research librarian.  NAP is a group of 49 individuals from across Canada  (2 pharmacists; 1 psychologist) | The Canadian Guideline is intended to assist physicians with decisions to initiate appropriate trials of opioid therapy for patients with chronic non-cancer pain, to monitor long-term opioid therapy, and to detect and respond appropriately to situations of opioid misuse including addiction. It was not designed to serve as a standard of care nor as a training manual. The document addresses safe and effective prescribing of opioids for CNCP (defined as pain that persists for more than six months) in male and female adolescents and adults. The target audience is primary-care physicians and medical and surgical specialists who manage patients with CNCP. Pharmacists, nurses, and dentists may also find it useful. The scope does not include using opioids for acute pain and end-of-life pain, or CNCP treatment modalities and approaches other than opioids. |
| **CPG 4**  **(RNAO)** | Expert panel = 13  Best Practice guideline team = 7  Stakeholder team = 28 | All registered nurses; 1 project coordinator; 2 medical doctors | This guideline provides evidence-based recommendations for nurses and other members of the inter-professional team who are assessing and managing people with the presence, or risk of, any type of pain. |
| **CPG 5**  **(SIGN)** | Development group = 23  Reviewers = 16  Editorial group = 7 | 7 medical doctors of varying speciality; 2 psychologists; 2 physiotherapists; 2 occupational therapists, 2 pharmacologists; 2 patient representatives; 1 each of the following: nurse, alternative health, evidence and information scientist, program manager. | This guideline provides recommendations based on current evidence for best practice in the assessment and management of adults with chronic non-malignant pain in non-specialist settings.  This guideline will be of particular interest to all healthcare professionals involved in the assessment and management of patients with chronic pain, including general practitioners, pharmacists, anaesthetists, psychologists, psychiatrists, physiotherapists, rheumatologists, occupational therapists, nurses, patients, carers and voluntary organisations with an interest in chronic pain. |
| **CPG 6**  **(UM)** | 5 + 3 consultants | 1 medical educationalist; 1 rehabilitation medicine, 9 medical doctors of varying speciality | To provide a systematic framework for providers to evaluate and manage patients with chronic, non-terminal pain with special attention to specific principles of opioid management. |
| **CPG 7**  **(WSAMDG)**  **interagency** | There is a list of 27 advisors | Unable to locate. | It is intended as a resource for primary care providers treating patients with chronic non-cancer pain. It does not apply to the treatment of acute pain, cancer pain, or end-of-life (hospice) care. |
| **CPG 8**  **(AP -AAP)** | 21 | 12 medical doctors with varying specialities; 3 registered nurses; 1 pharmacologist, 1 psychologist; 1 bioethics; 1 health policy; 1 physician assistant | The intent of the guideline is to provide evidence based recommendations for the use of chronic opioid therapy for chronic non-cancer pain in both primary and speciality settings. The target audience is clinicians who provide care for patient with chronic non-cancer pain including cancer survivors with chronic pain due to their cancer or its treatment. Management of cancer pain, pain at the end of life, acute pain, post-surgical pain, labour pain or chronic non-cancer pain in children or adolescents is outside the scope of this guideline |
| **CPG 9**  **(Philadelphia Panel)** | Clinical speciality experts = 10  Ottowa methods group = 7 | 8 medical doctors with varying specialities; 2 physiotherapist;  1 physiotherapist; 1 nurse; 1 information scientist; 1 medical doctor; 3 epidemiologists | Purpose of the group was to create evidence-based practice guidelines that identify the clinical benefit of rehabilitation interventions for low back, knee, neck, and shoulder problems, the guidelines did not address medical or pharmacological management of these conditions. Although the guidelines primarily benefit the rehabilitation specialist (physical therapists, occupational therapists, and sports therapists), family practitioners and other primary care physicians are responsible for managing these conditions and their treatments. By knowing which rehabilitation interventions have proven clinical benefit, physicians can better coordinate a patient's care and make evidence-based decisions when ordering physical therapy. |
| **CPG 10**  **(Raff)** | 6 | Medical doctors with varying specialities | To provide a brief and practical guideline for the use of chronic opioid therapy in patients with chronic non-cancer pain. The target audience is all clinicians in primary and specialty settings who provide care for adults suffering from chronic non-cancer pain. The management of acute pain, postsurgical pain, labour pain, cancer pain, pain at the end of life, and chronic non-cancer pain in children is not addressed in this guideline. |
| **CPG 11**  **(Saunders et al )** | 3 | 1 medical doctor; 2 unclear | The need for appropriate evidence-based practice guidelines to effectively treat this most impaired and dysfunctional segment of chronic pain patients continues to be high. This article offers a second evidence-based update to treatment guidelines for chronic non-malignant pain syndrome patients. |
| **CPG 12**  **(Schnitzer)** | 1 | Medical doctor | This paper reviews the evolution of pain management guidelines in response to the availability of COX-2selective anti-inflammatory drugs, before introducing updated guidance from the Working Group on Pain Management, which has been collated in response to recent developments concerning COX-2 inhibitors and other NSAIDs. |
