# Supplementary material for: Clinical practice guidelines for the management of chronic musculoskeletal pain in primary healthcare: a systematic review
Source: Implement Sci. 2017 Jan 5;12:1. doi: 10.1186/s13012-016-0533-0 (PMC5217556; doi:10.1186/s13012-016-0533-0)
Supplement: Additional file 2: — More on DOMAIN 5: applicability. (DOCX 17 kb) [file 13012_2016_533_MOESM2_ESM.docx]

**Additional file 2_More on DOMAIN 5: Applicability**

|  |  | **Decision process** | **18. Barriers and facilitators to application** | **19. Advice/Tools to put recommendations to practice** | **20. Potential resource limitations have been considered** | **21. Monitoring and audit criteria** |
| --- | --- | --- | --- | --- | --- | --- |
| **CPG 1** | ASIPP 2012 | Consensus process | They gave information on what the provider would need to implement in terms of staff and patients | Algorithms for implementation  Opioid tools | Not clear | Section on monitoring. The need for monitoring of the implementation of recommendations is emphasised. Important for opioid prescription and adherence monitoring.  Policy |
| **CPG 2** | ICSI 2013 | Consensus process  Consensus group used their experience about the context | The work group gives consideration to the importance of many issues as they develop the guideline. These considerations include the systems of care in our community and how resources vary, the balance between benefits and harms of interventions, patient and community values, the autonomy of clinicians and patients and more. All decisions made by the work group are done using a consensus process | Resource toolkit | Those that did well included reference to potential resource implementation. | Separate Implementation document with implementation strategies and quality improvement as well as for outcome reporting  Policy |
| **CPG 3** | NOUGG 2010 | Consensus process | Some are mentioned. E.g. systemic barriers exist that could reduce compliance.  Implementation efforts should include raising awareness with multiple-system stakeholders  about the role they can play in improving the effectiveness and safety of opioid prescribing. | Roadmap/flow diagram  Appendices = Tools  Patient resources, toolkit | Some are considered for drug screening | Some info on monitoring /evaluation  Policy – prescribing and monitoring opioids |
| **CPG 4** | RNAO 2013 | Consensus development | Other factors that will affect the use of this guideline include each organization’s policies and procedures, government legislation, different health-care sectors and the client population. It is important to acknowledge that personal preferences and unique needs, and the resources available | Implementation toolkit | Advises more research on health systems – e.g impact education electronic assessment | Yes clear criteria  Policy |
| **CPG 5** | SIGN 2013 | Consensus  DECIDE EtD framework  In handbook | Individualised implementation plan | Yes  Patient resources  Tools – outcome measures | Yes – considered Scottish context  Referred to cost -effectiveness where indicated.  Budget impact is a criterion in the decision making process: DECIDE (not mentioned in CPG document).  Advise on more research about cost-effectiveness. | Context specific  Separate implementation plan |
| **CPG 6** | UMHS 2011 | Consensus | Considered side effects | Patient and staff resource material  Training manual  Assessment and screening tools | Included the prices of the medication | Monitoring tools for opioid  Policy |
| **CPG 7** | WSAMDG 2010 | Expert consensus | Not clear | Tools and algorithm  Clinical tools and resources  Patient information | Not clear | Monitoring and evaluating the use and efficacy of opioids |
| **CPG 8** | APS AAP 2009 | Consensus (Delphi) | Mentions barriers to interdisciplinary care – e.g. high cost  Others are inferred. | Screening tool | Not explicitly, but as part of the recommendation discussion.  Used GRADE | Yes – although recommendation specific.  Monitoring opioids use; clinicians should be aware of local policies regarding prescription |
| **CPG 9** | Harris & Susman 2002 | Expert panel | Self-stated as poor | - | Not clear | Not clear |
| **CPG 10** | Raff et al. 2014 | Consensus | Not clear | Algorithm_ assessment tools screening | Not clear | Not clear |
| **CPG 11** | Sanders et al. 2005 | Not clear | Some – specific to work conditions;  Return to work. | Algorithm | Not clear | Not clear |
| **CPG 12** | Schnitzer 2006 | Not clear | Not specific | Algorithm | Not clear | Not clear |
